# Antarctic grounding line delineation from the Italian Space Agency COSMO-SkyMed DInSAR data

**DOI:** 10.1038/s41597-025-06023-3

**Published:** 2025-11-03

**Authors:** Natalya Ross, Pietro Milillo, Luigi Dini

**Affiliations:** 1 Department of Civil & Environmental Engineering, University of Houston, Houston, TX USA; 2 Department of Earth & Atmospheric Sciences, University of Houston, Houston, TX USA; 3 German Aerospace Center (DLR), Microwaves and Radar Institute, Munich, Germany; 4 Italian Space Agency (ASI), Matera, Italy

**Keywords:** Cryospheric science, Cryospheric science

## Abstract

This paper presents an Antarctic grounding line dataset, manually mapped using Differential Interferometric Synthetic Aperture Radar (DInSAR) data from the COSMO-SkyMed X-band radar satellite mission. The dataset comprises 794 double difference interferograms with corresponding grounding line products. The data has been collected over 74 glaciers in East Antarctica, West Antarctica, and the Antarctic Peninsula between July 2020 and March 2022. Each DInSAR interferogram was generated using two pairs of radar images, with a one-day interval between images in each pair and acquisition intervals between pairs ranging from 16 to 64 days. The dataset, which relies solely on COSMO-SkyMed data and leverages 1-day repeat-pass interferometry, enables precise grounding line mapping in fast-flowing regions, where sensors like Sentinel-1 and ICESat-2 encounter limitations. This dataset provides extensive coverage across Antarctica and enables the observation of grounding line migrations driven by ocean tides. Furthermore, compared to previously available datasets, it allows for the estimation of long-term retreat rates for several glaciers, including Thwaites, Pine Island, Totten, and Moscow University glaciers.

## Background & Summary

Antarctica is a significant contributor to global sea level rise, with the potential to substantially increase the mean sea level by the end of this century^1–3^. Continuous monitoring of Antarctic evolution is important to understand ice sheet dynamics, minimizing uncertainties in sea level rise projections, and develop strategies to mitigate the risks posed by rising sea levels^4–6^. This monitoring can be achieved by tracking the position of the grounding lines, which mark the boundaries where Antarctic glaciers detach from the bedrock and begin to float in the ocean^7,8^. Grounding lines play a fundamental role in controlling glacier force and mass balances, making them a critical component for understanding glacier dynamics and a key indicator of glacial stability^9–14^. Inland grounding line retreat results in increased mass loss, highlighting grounding line importance in monitoring glacier contribution to sea level rise caused by climate warming^15–18^. Therefore, accurate grounding line delineation and continuous monitoring of their location is essential for investigating glacier stability and projecting future sea-level changes^19–21^.

Numerous publicly available grounding line datasets have been acquired over various Antarctic glaciers. Among these, the MEaSUREs dataset^22^, which compiles data obtained from 1992 to 2014 by ERS, RADARSAT, ALOS PALSAR, and Sentinel-1 satellite missions, is the most extensive. Despite its wide coverage, the MEaSUREs dataset^22^ omits some glaciers, such as the main trunk of the Dibble glacier, and the Astrolabe glacier. Furthermore, some glacial data is available only for select years, often limited to a single or a few observations.

More recently, localized grounding line datasets have been introduced, offering high-resolution observations for specific regions. For example, Milillo *et al*. provide grounding line data for the Amundsen Sea Embayment between 2016 and 2020^23^, while Wallis *et al*. focus on the grounding lines in Antarctic Peninsula from 2019 to 2020^7^. Although these datasets enhance regional studies, their limited spatial coverage does not allow comprehensive Antarctic-wide studies.

In 2021, two global grounding line Antarctic datasets became available. The first dataset, released by the ESA, contains updated grounding line records for key Antarctic glaciers derived from ERS-1/2, TerraSAR-X, and Copernicus Sentinel-1 data collected between 1994 and 2020^24^. While this dataset extends the temporal range of observations compared to the MEaSUREs dataset, it still lacks coverage for several glaciers, such as Larsen D and George VI ice shelfs, Rennick, Dibble, Veststraumen, Stancomb-Wills, Hull, and Land glaciers, and the main trunk of the Bailey glacier. Similar to MEaSUREs, some regions are represented by a limited number of grounding line measurements. As a result, despite its expanded timespan ending in 2020, the ERA’s dataset does not provide the comprehensive coverage required for continuous monitoring of Antarctic evolution. The second dataset^25^, presented in Mohajerani *et al*. (2021), was generated exclusively from the Sentinel-1 interferograms, acquired in 2018, while the grounding line mapping was performed using a deep learning approach and subsequently verified by human experts. While it offers broad coverage of almost the entire Antarctic coastline, it omits certain areas, such as the main trunks of Dibble, Robert, and Wilma Glaciers, or the Slessor and Bailey Glaciers.

In 2022, another global Antarctic dataset was published, presenting flexure zone products derived from the ICESat-2 laser altimetry mission^26^. This dataset includes points corresponding to the landward limit of ice flexure (hinge line), the break in surface slope, and the seaward limit of ice flexure, extracted along ICESat-2 satellite tracks. While the dataset offers global coverage, it provides single-point grounding line locations, each acquired at different times of the year, rather than a continuous grounding line record. Consequently, it is limited in its ability to depict the complete grounding line along the main trunk of a glacier, offering only discrete points along the grounding line.

Over the years, various techniques have been developed for mapping grounding lines, including hydrostatic methods^27–32^, surface slope methods^33–38^, repeat-track laser altimetry^39^, pseudo crossover radar altimetry^40^, Synthetic Aperture Radar (SAR) differential range offset tracking^41^, and Differential Interferometric SAR (DInSAR)^42–44^. While each method has distinct advantages and limitations^45^, DInSAR stands out for its ability to operate under all weather conditions, and its proven effectiveness in continuously monitoring grounding lines and detecting their rapid migrations^46–49^.

The DInSAR technique enables grounding line mapping with an accuracy of approximately 100–200 meters^50,51^. This method involves combining three to four SAR images acquired at different times over the same area and extracting the grounding line position from the interferometric fringes^52^. However, when ice properties change or glacier displacement exceeds the radar’s range of detection due to an unsuitable combination of radar wavelength and satellite revisit interval, decorrelation and aliasing occur, making grounding line mapping impossible^53–55^. For instance, the 6-day repeat pass and 5.6 cm wavelength configuration of ESA’s Sentinel-1A/B mission is insufficient for observing grounding lines of fast-flowing glaciers, such as the main trunks of Totten and Denman glaciers or the glaciers in the Amundsen Sea Embayment^47,49,56^. In contrast, the COSMO-SkyMed (CSK) constellation operated by the Italian Space Agency, which uses X-band radar (3 cm wavelength) and a 1-day repeat pass, produces coherent DInSAR signals even for fast-flowing Antarctic glaciers, effectively overcoming the limitations of Sentinel-1a/b^42,51,52^. Therefore, while C-band interferometry with a 6-day repeat pass is suboptimal for mapping Antarctic grounding lines, X-band interferometry with a 1-day repeat pass has proven to be the most effective configuration for accurate grounding line mapping, even over fast-moving regions^48,52^.

Here, we present a CSK DInSAR dataset acquired over major Antarctic glaciers between July 2020 and March 2022, along with the corresponding grounding lines, manually mapped from these data. Since the CSK constellation does not operate under an open data policy, the primary motivation for producing this dataset is to expand the availability of freely accessible grounding lines to the entire scientific community. Unlike previously available datasets with global coverage, which often combine data from multiple satellite missions, the dataset presented here is derived exclusively from the CSK mission.

We analyze the seasonal variations in interferogram coherence and their impact on the accuracy of grounding line delineation. Additionally, we compare the CSK-derived grounding lines with previously published datasets, identifying similarities and differences. We also assess long-term glacier retreat rates since 1996, identifying stable glaciers as well as those exhibiting significant retreat of up to 700 m/year. Furthermore, we highlight specific glaciers where our dataset provides unique coverage not available in other public datasets, emphasizing its importance in filling critical gaps in Antarctic grounding line observations.

## Methods

### COSMO-SkyMed satellite mission

**Fig. 1:**
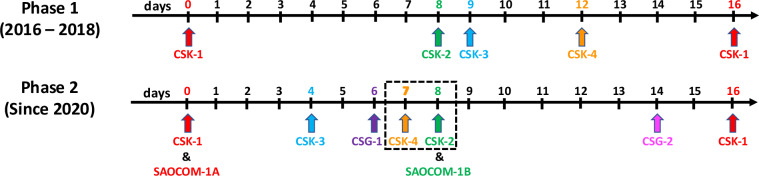
CSK satellite configuration. The images, acquired by the second phase CSK-2 and CSK-4 satellites, highlighted with a black frame, were used here to generate DInSAR interferograms.

COSMO-SkyMed (CSK) is a low Earth orbit satellite mission operated by the Italian Space Agency (ASI) at an altitude of approximately 620 km. Each satellite in the constellation has a 16-day repeat cycle and is equipped with an X-band Synthetic Aperture Radar (SAR) antenna operating at a wavelength of 3.1 cm or a frequency of 9.6 GHz. The first generation of the CSK constellation, launched between 2007 and 2010, consisted of four identical satellites (CSK-1, CSK-2, CSK-3, and CSK-4). The satellites were offset in their orbits to provide irregular yet consistent acquisition intervals, including eight days between CSK-1 and CSK-2, one day between CSK-2 and CSK-3, three days between CSK-3 and CSK-4, and four days between CSK-4 and CSK-1^57^ (Fig. 1).

Two second-generation satellites, CSG-1 and CSG-2, were launched in 2019 and 2022, respectively, into the same orbit as the COSMO-SkyMed (CSK) satellites. Additionally, in collaboration with Argentina’s Space Agency (Comisión Nacional de Actividades Espaciales, CONAE), two L-band SAOCOM satellites, SAOCOM-1A and SAOCOM-1B, were introduced to the COSMO-SkyMed orbit in 2018 and 2020^58^. After the CSG and SAOCOM launch, the satellite orbital offset started providing the following acquisition intervals between the satellites: four days between CSK-1 and CSK-3, two days between CSK-3 and CSG-1, one day between SCG-1 and CSK-4, one day between SCK-4 and SCK-2, six days between CSK-2 and CSG-2, and two days between CGS-2 and CSK-1^51^. The acquisition time of SAOCOM-1A closely matches the acquisition time of CSK-1, while SAOCOM-1B shares the same acquisition day as CSK-2 (Fig. 1).

CSK is a unique and comprehensive satellite mission as it provides a variety of acquisition modes, all possible combinations of transmitted and received signal polarizations, and both right- and left-looking acquisition geometries, along with ascending and descending acquisition directions. While the nominal acquisition geometry for CSK is right-looking, the platform’s motility also enables left-looking imaging mode. CSK supports three operational acquisition modes: Spotlight (high resolution with a small coverage area), Stripmap (medium resolution with medium coverage area), and ScanSAR (coarse resolution with a large coverage area). Among these, Stripmap Mode was selected for this study as it offers an optimal balance between spatial resolution and coverage area. In Stripmap Mode, the satellite antenna maintains a constant angle relative to the platform’s flight direction, enabling it to scan a continuous strip on the illuminated surface as the platform moves.

### COSMO-SkyMed data processing

All SAR acquisitions utilized in this study were performed in horizontal transmit and horizontal receive mode (HH), which has been shown to provide the highest signal-to-noise ratio (SNR) for glacier application^59,60^ (Fig. 1). All the SAR scenes were delivered by the Italian Space Agency (ASI) and analyzed in the single-look complex (SLC) format, meaning that the radar signal is provided in the form of complex numbers, combing both amplitude and phase information. In Stripmap-HIMAGE mode, the CSK data is sliced along the satellite track into consecutive overlapping frames, each 40 km in length, with a 10 km overlap, ensuring a consistent 40-km swath in the azimuth direction. These frames are also characterized by a swath width of 40 km in range (cross-track) and a 3-m single-look spatial resolution in both azimuth and range.

**Fig. 2:**
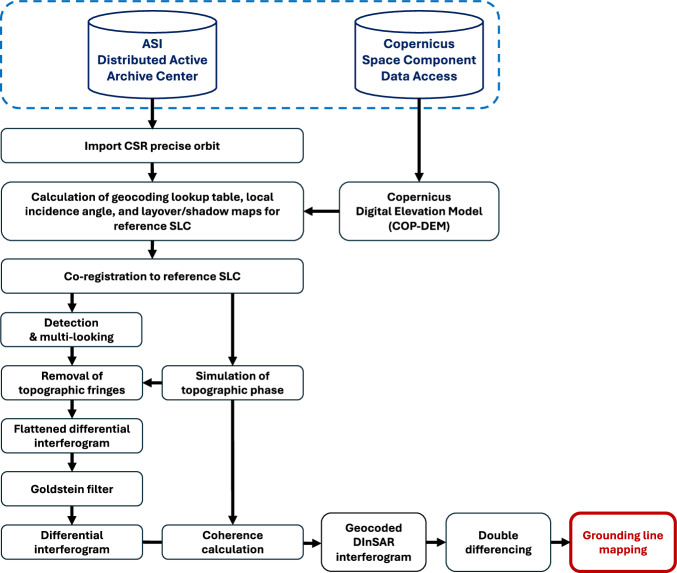
CSK data processing algorithm. Goldstein Filter is referred to the Goldstein and Werner phase filter, which reduces noise in the interferometric phase while preserving fringe patterns^67^.

Data processing was carried out using GAMMA software, with the processing workflow schematics shown in Fig. 2. The data were acquired along 156 CSK orbital tracks. For each track, processing began with the conversion of SAR scenes from SLC format to GAMMA format. Subsequently, between 3 and 9 consecutive overlapping frames were combined into a single SAR image, depending on the glacier observed. The number of frames was determined based on the size of each glacier and was tailored individually in collaboration with ASI to ensure optimal coverage for each glacier.

After importing the precise CSK orbit data, this information, along with Copernicus Digital Elevation Models (DEMs), were utilized to generate look-up tables, local incidence angle maps, and layover/shadow masks. The look-up tables provide the transformation functions required to convert slant-range geometry into coordinates in the EPSG:3031 Polar Antarctic Stereographic Projection. The accuracy of these transformation functions was validated using an intensity cross-correlation method. This method involves simulating a radar backscatter image using the Copernicus DEM, based on assumptions how radar backscatter intensity varies with terrain topography. The CSK radar images were divided into small sections (image chips), and each chip was matched against the simulated reference image. The range and azimuth offsets of these matches were measured. A polynomial regression fit was then applied to the offsets, and the standard deviations of the offsets from the fitted curve were calculated for quality control purposes.

Co-registration to the reference SLC was carried out using co-registration look-up tables generated from the precise orbit data and the DEM, which was resampled to the slant-range geometry of the reference SLC. A multi-looking factor of 16 in both range and azimuth was applied in both the azimuth and range directions, resulting in an interferogram resolution of 48 m × 48 m.

**Fig. 3:**
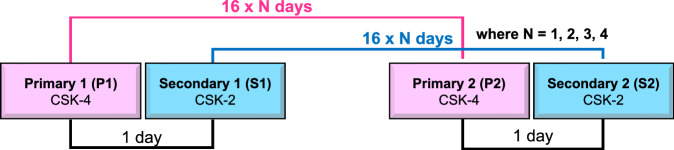
Schematic representation of a DInSAR interferogram generation process.

To generate a double-difference DInSAR interferogram, we used two pairs of SAR images. In each pair, the first image (primary image, P) was acquired by CSK-4, and the second image (secondary image, S) was collected by CSK-2, with a one-day interval between them (Fig. 3). The acquisition interval between the pairs, defined as the interval between the two primary images, P1 and P2, is determined as 16⋅N days, where 16 is the satellite revisit interval. The shortest interval analyzed was 16 days (N = 1), and the longest was 64 days (N = 4). The DInSAR interferogram generation process reveals the vertical motion of a glacier. Each DInSAR fringe corresponds to half the radar wavelength of vertical displacement in the satellite’s line-of-sight.

**Fig. 4:**
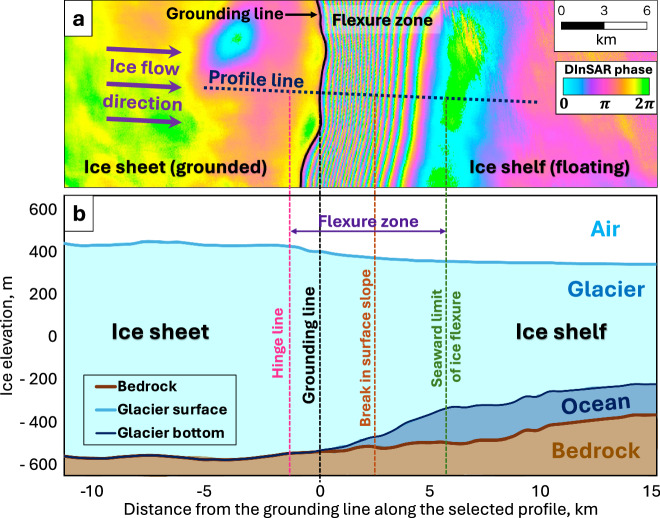
Visualization of grounding mapping process: (**a**) DInSAR-based grounding line delineation; (**b**) correspondence of the DInSAR interferogram to the glacier geometry. Bed and surface profiles were retrieved from BedMachine Antarctica^68^ along the selected profile. The bottom surface of the glacier is unknown and was hypothesized for illustrative purposes.

For the X-band radar, this allows the detection of 1.5 cm displacement per fringe in the satellite’s line-of-sight or approximately 1.7 cm of vertical surface displacement when projected vertically keeping into account the CSK look angle. The grounding line position can be identified from the DInSAR interferogram as the most inland fringe with an accuracy of 100–200 m^44,48,52^ (Fig. 4).

### Grounding line mapping using COSMO-SkyMed data

The flexure zone, where the glacier transitions to flotation, is located immediately seaward of the grounding line and is represented by DInSAR fringes on a double-difference interferogram. Both the floating ice shelf, located seaward of the interferometric fringes, and the grounded ice sheet, situated inland of the flexure zone, appear as fringe-free areas on a DInSAR interferogram. Consequently, the grounding line can be manually delineated as the most inland fringe where vertical glacier displacement is observed (Fig. 4). The time-intensive process of grounding line mapping was carried out using the freely available QGIS software^61^, requiring approximately 900 hours of work by the primary operator and an additional 200 hours for a second operator to verify the results.

Ocean tides cause continuous variations in glacier surface elevation throughout the tidal cycle. As a result, each of the four SAR images used to produce a DInSAR interferogram corresponds to a specific tidal level at the time of image acquisition. The number of fringes in an interferogram represents the difference in tidal levels along the radar’s line of sight across the four SAR images^52^, which ensures that the number of fringes between the grounded and floating ice remains consistent within each interferogram. However, the extent of water penetration beneath the glacier can vary due to factors such as bedrock slope, glacier thickness, and tidal levels. As a result, tidal deformation may extend farther inland in certain sections along the grounding line, leading to larger fringe spacing within the flexure zone in some areas. Therefore, we carefully monitor the number of tidal fringes during the mapping process to prevent misplacing the grounding line. Although fringe spacing in the flexure zone may increase in specific regions, the total fringe count between the grounded and floating ice remains constant. This consistency provides a reliable metric for accurate grounding line identification. In the presence of active subglacial water, the innermost interferometric fringe may become diluted and spread out. This can make it difficult to determine whether the glacier is floating or grounded with subglacial activity occurring beneath it. The grounding line mapping technique we used, namely tracking the number of fringes within an interferogram, helps reduce the risk of misplacing the grounding line and minimizes the likelihood of misinterpreting zones of subglacial activity as the grounding line.

## Data Records

**Fig. 5:**
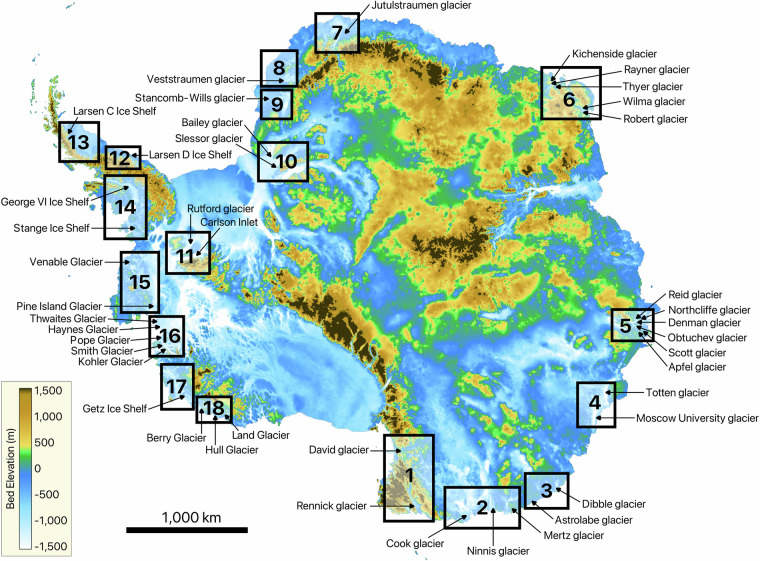
Locations of the 18 areas, where the analyzed glaciers are located.

**Fig. 6:**
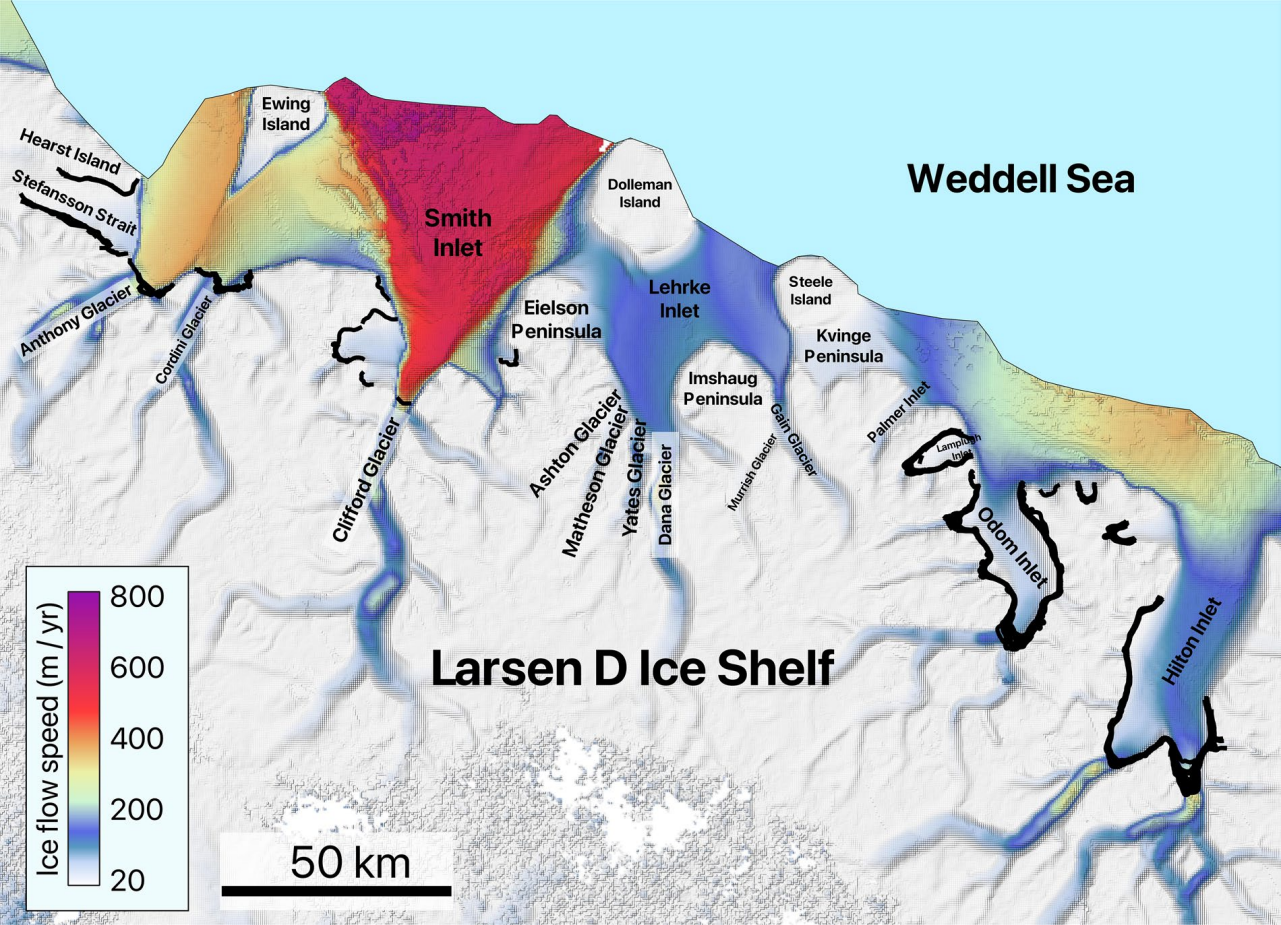
Detailed view of area 12, showing the names of the glaciers located in the area along with all the manually mapped grounding lines. The second version of the MEaSUREs InSAR-based ice velocity map^69^, used as the background here, is displayed in the EPSG: 3031 projection.

**Fig. 7:**
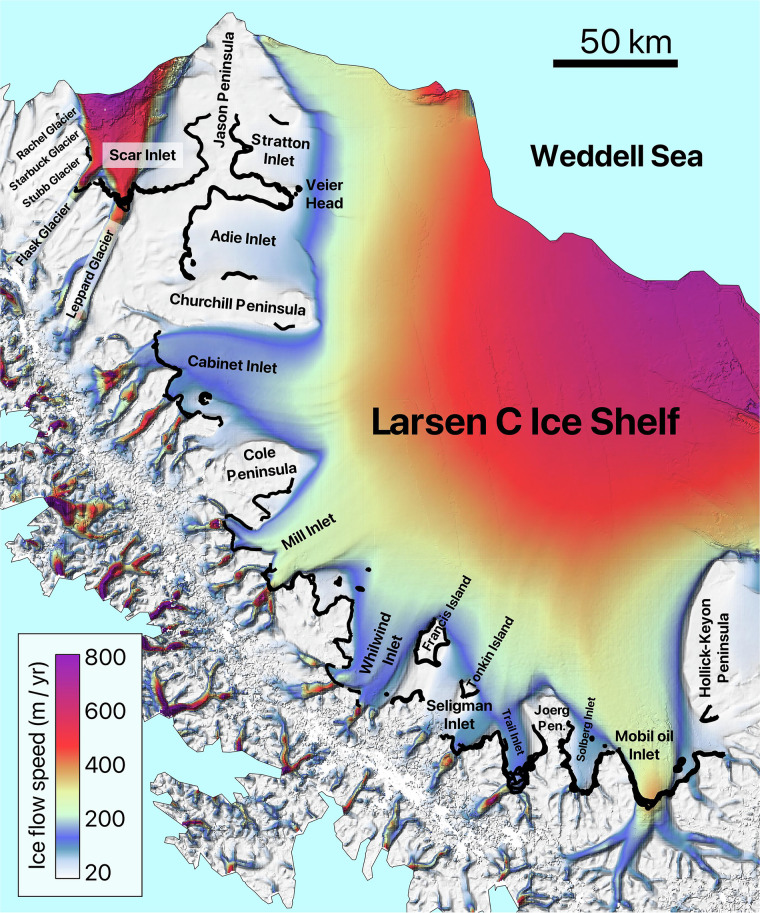
Detailed view of area 13, showing the names of the glaciers located in the area along with all the manually mapped grounding lines. The second version of the MEaSUREs InSAR-based ice velocity map^69^, used as the background here, is displayed in the EPSG: 3031 projection.

**Fig. 8:**
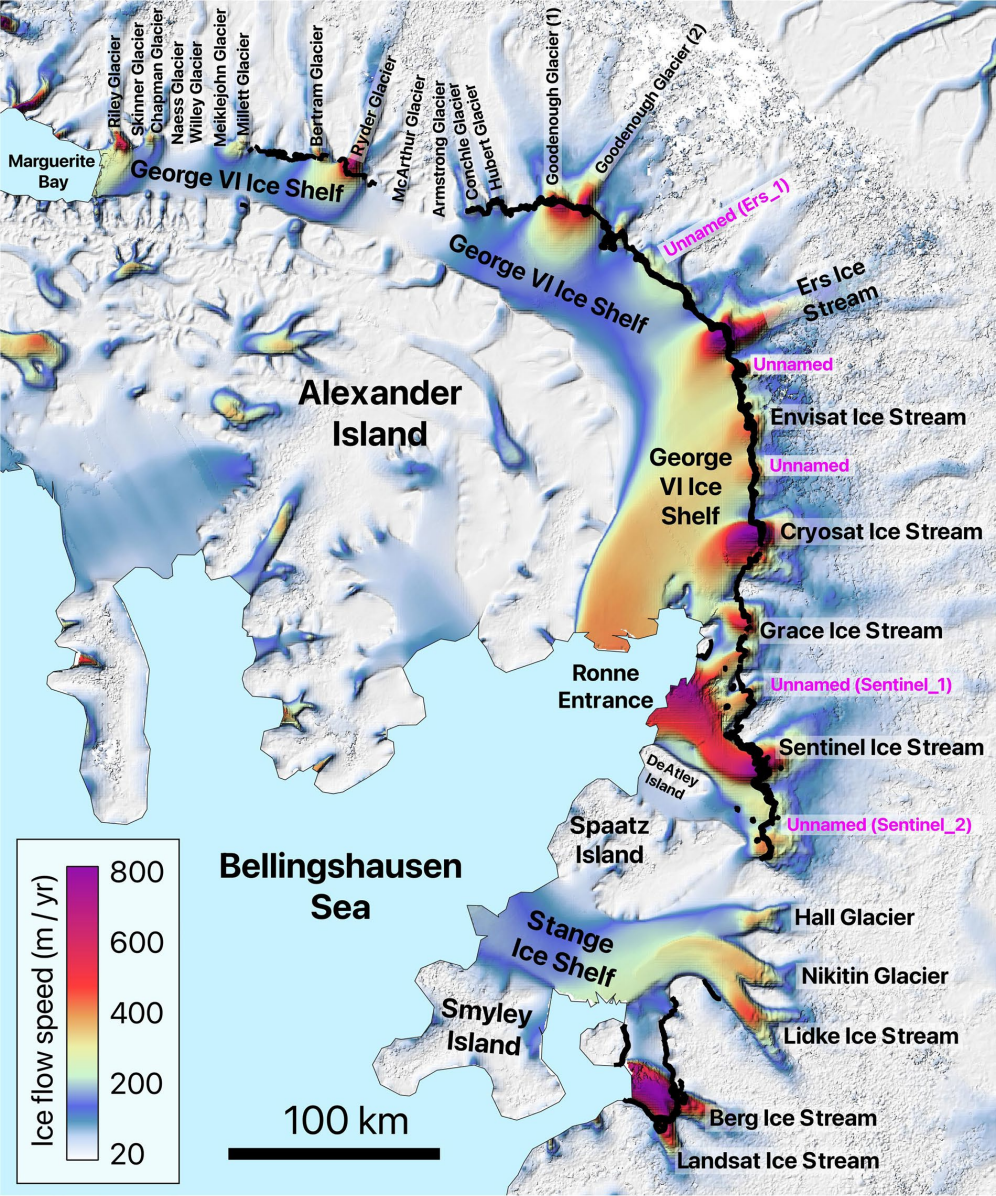
Detailed view of area 14, showing the names of the glaciers located in the area along with all the manually mapped grounding lines. The second version of the MEaSUREs InSAR-based ice velocity map^69^, used as the background here, is displayed in the EPSG: 3031 projection.

**Fig. 9:**
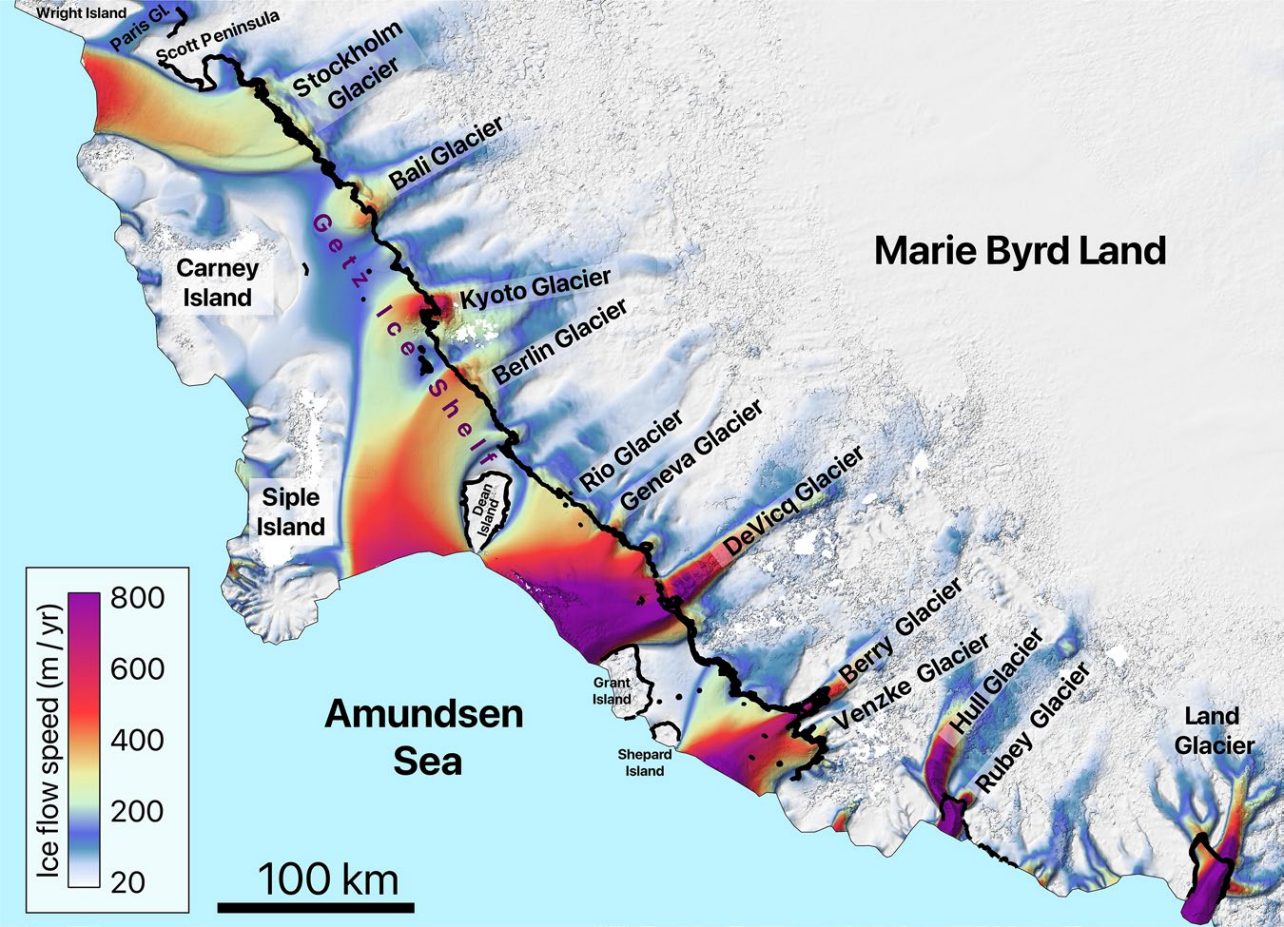
Detailed view of areas 17 and 18, showing the names of the glaciers located in the area along with all the manually mapped grounding lines. The second version of the MEaSUREs InSAR-based ice velocity map^69^, used as the background here, is displayed in the EPSG: 3031 projection.

The dataset is available at Figshare (Reference number: 28459139)^62^. The DInSAR interferograms used for manual grounding line mapping cover a wide range of Antarctic glaciers distributed across the continent (Fig. 5). To organize the analyzed glaciers, we divided them into 18 geographic regions based on their locations. The boundaries of these areas, along with the names of the glaciers within each, are shown in Fig. 5. For areas 12, 13, 14, and 17, only the corresponding ice shelf names are displayed in Fig. 5. Detailed zoomed-in views of these regions, including individual glacier names and all manually delineated grounding lines, are provided in Figs. 6,7,8,9, respectively.

The publicly available dataset presented in this paper comprises two primary directories: ‘grounding lines’ and ‘interferograms’:

- The ‘grounding lines’ directory contains a single shapefile, ‘CSK_grounding_lines_2020-2022_v0.1.shp,’ which consolidates all manually delineated grounding lines from the study period between July 2020 and March 2022.
- The ‘interferograms’ directory includes the phase and coherence of all the available interferograms. These files are organized into 18 subdirectories named ‘area_01’ to ‘area_18,’ corresponding to the 18 defined regions, shown in Fig. 5.

The naming convention for the interferograms is as follows:

- For DInSAR coherence files: ‘XX_cocoP1_S1-P2_S2.flat.topo_off.psfilt.geo.coh.tiff’;
- For DInSAR phase files: ‘XX_cocoP1_S1-P2_S2.flat.topo_off.psfilt.geo.tiff’.

Here, XX refers to the area code (ranging from 01 to 18), and P1, S1, P2, S2 denote the primary and secondary acquisition dates of the first and second pair of images, respectively. The dates are formatted as ‘YYYYMMDD’.

The ‘CSK_grounding_lines_2020-2022.shp’ shapefile includes the following attributes:

1. Area: Region (ranges from 1 to 18) where the glacier is located an (Fig. 5).
2. Glaciers: Names of the glaciers covered by the corresponding interferogram (Fig. 5).
3. Land: Antarctic region where the glacier is situated, including Victoria, George V, Wilkes, Enderby, Dronning Maud, Coats, Ellsworth, Graham, and Marie Byrd Lands.
4. Location: Classification of the glacier’s location as East Antarctica, West Antarctica, or the Antarctic Peninsula.
5. Primary1: Acquisition date of the first image in the DInSAR interferogram (in YYYYMMDD format).
6. Secondary1: Acquisition date of the second image in the DInSAR interferogram (in YYYYMMDD format).
7. Primary2: Acquisition date of the third image in the DInSAR interferogram (in YYYYMMDD format).
8. Secondary2: Acquisition date of the fourth image in the DInSAR interferogram (in YYYYMMDD format).
9. DD: Double difference interferogram name, based on which the corresponding grounding line was mapped (formatted as Primary1_Secondary1-Primary2_Secondary2).
10. Revisit: Revisit interval between the SAR image pairs (multiples of 16 days, ranging from 16 to 176 days).
11. Time: Time of SAR image acquisition (in HHMMSS format).
12. Coherence: Average coherence of the corresponding interferogram.

The DInSAR dataset consists of 794 pairs of phase and coherence data, covering multiple glaciers across the Antarctic Peninsula, as well as both East and West Antarctica (Fig. 5).

The details of the analyzed DInSAR dataset are summarized in Table 1, which outlines the primary glaciers covered by the interferograms and their distribution across the designated areas. The table includes the number of interferograms covering each glacier. For regions 12, 13, 14, and 17, Table 1 lists only the ice shelves, while the individual glaciers within these ice shelves are identified in Fig. 6, Fig. 7, Fig. 8, and Fig. 9, respectively. While the dataset covers the main trunks of most glaciers in regions 12, 13, 14, and 17, some glaciers are not covered by the CSK satellites due to the orbital configuration of the mission. Additionally, Table 1 provides a grounding line measurement comparison between our dataset and the grounding line data from the MEaSUREs and ESA’s datasets. The grounding line counts for the MEaSUREs and ESA’s datasets, shown in Table 1, refer specifically to the number of grounding lines mapped along the main trunk of each glacier. Grounding lines mapped along glacier flanks, if present, were excluded from this table, as they do not provide significant insights into glacier retreat or tidally induced short-term glacier dynamics.

**Table 1:** List of glaciers and ice shelves covered by the analyzed DInSAR Interferograms.

Area	Total #	Glacier / Ice Shelf	Location	CSK	MEaSUREs	ESA
1	44	David	East	9	1 (1996)	1 (2016)
		Rennick	East	35	1 (2000)	NO
2	21	Cook	East	11	1 (1996)	2 (2017)
		Mertz	East	3	1 (1996)	NO
		Ninnis	East	7	1 (1996)	NO
3	30	Astrolabe	East	6	NO	NO
		Dibble	East	24	NO	NO
4	30	Moscow University	East	27	1 (1996)	1 (2017)
		Totten	East	3	2 (2013)	4 (2019)
5	11	Apfel, Denman, Northcliffe, Scott, Obtuchev, Reid	East	11	Denman glacier	
					3 (1996)	NO
6	23	Robert, Wilma	East	14	2 (2000)	NO
		Rayner, Thyer, Kichenside	East	9	2 (2000)	NO
7	60	Jutulstraumen	East	60	1 (1994)	1 (1994)
8	38	Veststraumen	East	38	2 (2000)	NO
9	32	Stancomb-Wills	East	32	3 (2000)	NO
10	46	Bailey	East	46	1 (2009)	NO
		Slessor	East		2 (2009)	1 (2014)
11	44	Carlson	West	12	2 (1995)	8 (2020)
		Rutford	West	32	2 (1996)	1 (1996)
12	20	Larsen D Ice Shelf*	Peninsula	20	Odom inlet	
					2 (1994)	NO
13	26	Larsen C Ice Shelf*	Peninsula	26	Trail Inlet	
					6 (1996)	4 (2017)
14	168	George VI Ice Shelf*, Stange Ice Shelf	Peninsula	168	Landsat Ice Stream	
					1 (1996)	NO
15	17	Venable	West	9	2 (1996)	1 (2017)
		Pine Island	West	8	7 (2011)	1 (1996)
16	45	Thwaites, Haynes, Pope, Smith East, Smith West, Kohler	West	45	Thwaites Butterfly	
					7 (2011)	1 (2016)
17	80	Getz Ice Shelf*	West	80	Kyoto glacier	
					1 (1996)	1 (2017)
18	59	Berry	West	35	1 (1996)	1 (2017)
		Hull	West	4	1 (1996)	NO
		Land	West	20	1 (1996)	NO

The ‘Total #’ column shows the total number of interferograms for each area, while the ‘CSK’ column lists the number of grounding lines per glacier analysed in study. The ‘MEaSUREs’ and ‘ESA’ columns show the number of grounding lines associated with the glacier’s main trunk and the most recent grounding line record in the corresponding datasets. Glaciers comprising the ice shelves, marked with an asterisk (*), are visualized in Fig. 6, Fig. 7, Fig. 8, and Fig. 9.

Approximately half of the glaciers analyzed in this study are missing from the ESA’s dataset, while most of the remaining glaciers include only a single grounding line record. This may be due to the TerraSAR-X coherence decorrelation, which is influenced by its 11-day repeat cycle intervals. In the MEaSUREs dataset, the majority of analyzed glaciers have only one historic grounding line, typically from the early 2000s or earlier, delivered from ERS-1, ERS-2 or ENVISAT. For example, our DInSAR dataset includes 35 grounding line records for the Rennick Glacier, whereas the MEaSUREs dataset contains only one grounding line, acquired in 2000 (25 years ago at the time of this publication) over its main trunk, and the ESA’s dataset does not include this glacier at all. For certain glaciers, our dataset provides grounding line records not available in either the MEaSUREs or ESA’s datasets. For example, the main trunks of the Astrolabe and Dibble glaciers are missing from both datasets but are covered in our dataset. In some cases, while the MEaSUREs or ESA’s datasets provide grounding line records for the main trunk of a glacier, our dataset fills data gaps. For instance, our dataset includes over 47 km of grounding lines for the Vennable Ice Shelf, which are missing in the MEaSUREs dataset. These examples highlight the wide coverage and significant value of our dataset in addressing limitations in existing datasets.

**Fig. 10:**
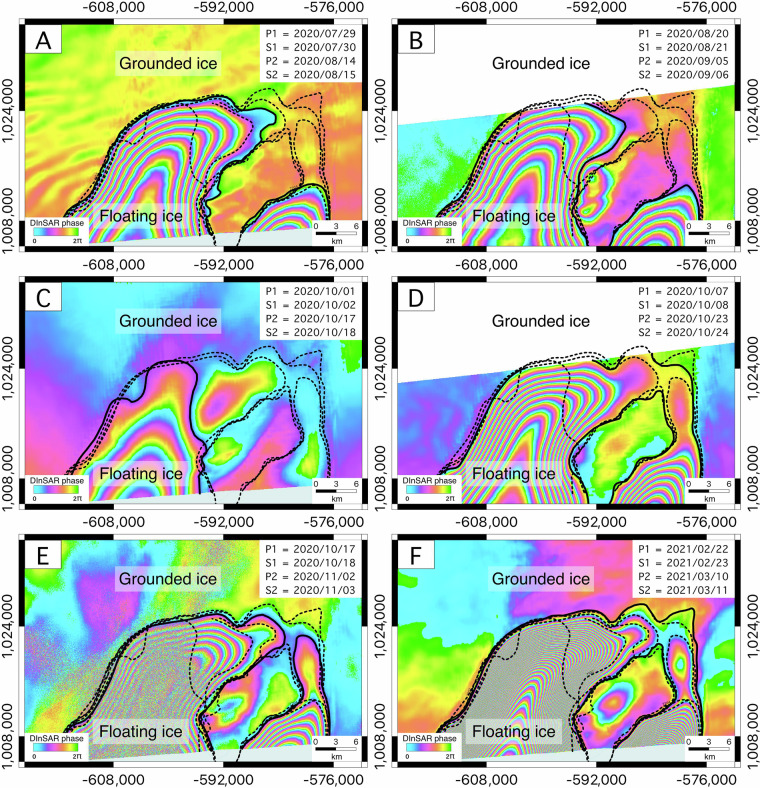
Tidally-induced short-term grounding line evolution of the Bailey Glacier, which is observable using our dataset. The interferograms are displayed in the EPSG: 3031 projection.

**Fig. 11:**
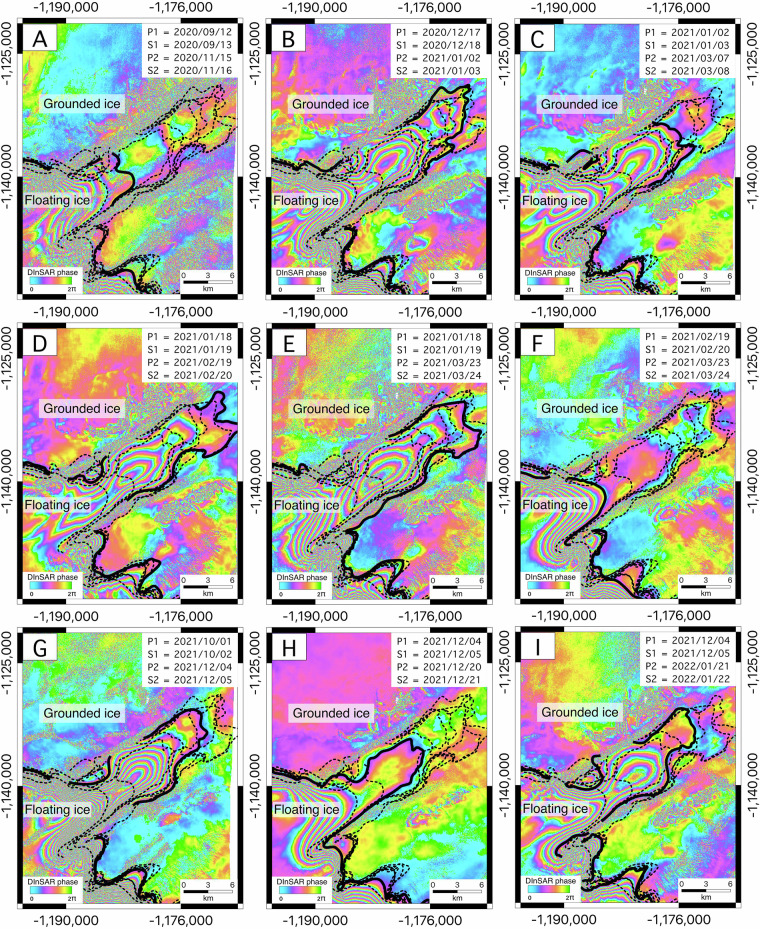
Tidally induced short-term grounding line evolution of the Berry Glacier, which is observable using our dataset. The interferograms are displayed in the EPSG: 3031 projection.

In addition to providing wide coverage of the major Antarctic glaciers (Fig. 5), the CSK DInSAR dataset allows the monitoring of tidal evolution of some of the considered glaciers. For example, the tidal signal is particularly evident for the Bailey (Area 10) and the Berry (Area 18) Glaciers. For the Berry Glacier, shown in Fig. 10, the subplots display sequential DInSAR interferograms arranged chronologically by acquisition date. These interferograms depict the back-and-forth movement of the grounding line over time, driven by tidal fluctuations. Initially separated interferometric fringes gradually move closer together and eventually merge, forming a fringe-free circular area between the connected fringes. This interferometric behavior provides an accurate representation of the actual dynamics of Berry Glacier. At high tide, ocean water lifts the glacier and intrudes beneath it through two distinct subglacial channels that eventually merge beneath the ice. At low tide, the water drains back through these channels, causing the glacier to settle downward, a cycle that repeats daily due to regular tidal fluctuations. Similarly, Fig. 11 illustrates the DInSAR interferograms for Berry Glacier, organized based on acquisition dates. These interferograms reveal the dynamic movement of the glacier’s main trunk grounding line, which undergoes a 19 km tidally-induced migration, advancing inland and retreating seaward under the influence of tidal forces. Therefore, our DInSAR dataset offers an opportunity to monitor and analyze tidal influences on glacier stability and grounding line dynamics.

## Technical Validation

**Fig. 12:**
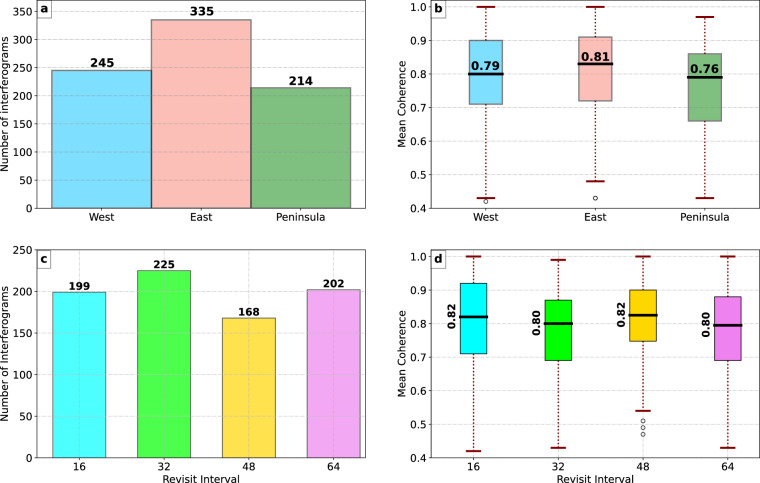
(**a**) Distribution of the interferograms by their geographic location; (**b**) Distribution of the mean DInSAR coherence by the interferograms’ location; (**c**) Distribution of the interferograms by the revisit intervals between the SAR pairs; (**d**) Distribution of the mean DInSAR coherence by the revisit intervals between the SAR pairs.

Combining 794 DInSAR interferograms, the dataset provides wide Antarctic coverage. Specifically, 245 interferograms cover glaciers in West Antarctica, with an average signal coherence of 0.79; 335 interferograms cover glaciers in East Antarctica, with an average signal coherence of 0.81; and 214 interferograms cover glaciers in the Antarctic Peninsula, with an average signal coherence of 0.76 (Fig. 12a,b). Here, the reported coherence values were calculated as the average coherence of the resulting 4-image DInSAR interferograms, which are provided in the dataset alongside the DInSAR phase. This dataset demonstrates a well-distributed coverage across all regions of Antarctica while maintaining consistently high coherence levels. The SAR image pairs used to produce double difference DInSAR interferograms were acquired at time intervals that are multiples of 16 days. The distribution of the available interferograms by their revisit intervals is illustrated in the subplot c of Fig. 12, which indicates that all the interferograms have a repeat cycle of 64 days or less.

All previously published datasets^22–26^ discussed earlier contain grounding line records acquired prior to the timeline of the dataset presented here, with MEaSUREs providing the earliest available grounding lines. Therefore, to assess the quality of our dataset, we calculate long-term retreat rates using the earliest record from MEaSUREs and compare them with more recent grounding lines from other datasets. In the MEaSUREs dataset^22^, the grounding line records from 1996 or the early 2000s lack the satellite acquisition time of the day. As a result, these historic grounding lines are often assumed to have been mapped at a zero-tide level, representing the average ocean height between high and low tides^47,48,52^. Consequently, while the MEaSUREs dataset does not support continuous monitoring of grounding line positions under varying tidal conditions, it remains valuable for evaluating long-term grounding line retreat in certain glaciers under the calm ocean assumption. For example, the grounding line positions of the Rennick, David, Ninnis, Vestraunem, Jutulstraumen, Hull, Stancomb-Wills, Carlson, and Rutford glaciers have remained stable over the past quarter-century. These observations are further supported by the deep learning-based grounding lines and the ICESat-2-derived point-wise grounding line locations^25,26^.

According to the MEaSUREs^22^, ESA’s^24^, and deep learning-based^25^ datasets, the grounding lines along the main trunks of the Denman, Rayner, Slessor, and Bailey glaciers have not exhibited significant or rapid long-term retreat since 1996. For example, as follows from MEaSUREs, over 24 years, between 1996 and 2020), the Denman glacier retreated at a rate of 140 ± 40 m/year, a value consistent with (Brancato *et al*.^49^). Using the other two datasets, the calculated retreat rates fall within the confidence interval of the MEaSUREs-derived value. Here, both the retreat rate and its standard deviation were calculated not at a single grounding line location, but as an average along the main trunk of the glacier. Multiple measurements, spaced 1 km apart, were taken along the glacier’s flowlines and then averaged to determine the mean grounding line retreat rate over the considered timeframe. The standard deviation calculation is calculated as the standard error of the mean for measurement with common uncertainty and accounts for an average error of 200 m in manual grounding line mapping, as reported in previous studies^44,52^. However, despite the relatively slow retreat, these four glaciers display large tidally induced grounding line migrations of several kilometers, which could expose them to water intrusion and basal melting^63,64^. Some glaciers exhibit a pronounced and measurable retreat. For instance, the main trunk of the Mertz Glacier retreated at a rate of 400 ± 20 m/year between 1996 and 2022, while the Venable Glacier retreated at 200 ± 10 m/year during the same period. Between 1996 and 2020, the eastern flank of the Cook Glacier remained stable, whereas its northern flank retreated at a rate of 410 ± 30 m/year. Similarly, the Berry Glacier experienced uneven retreat along its front. Between 1996 and 2021, the main trunk retreated at 440 ± 80 m/year, while the northern flank retreated at 260 ± 70 m/year. The higher uncertainty associated with Berry Glacier is attributed to the significant amplitude of tidally induced grounding line migrations. The flanks of the Land Glacier also retreated at different rates during the same period: the northern flank at 180 ± 30 m/year and the southern flank at 90 ± 20 m/year. Both Robert Glacier and Wilma Glacier displayed high tidal grounding line mobility, with Robert Glacier retreating at twice the rate of Wilma Glacier (320 ± 90 m/year vs. 160 ± 100 m/year) between 2000 and 2020. The retreat rates for Robert and Wilma glaciers cannot be verified using other datasets, as continuous grounding line records over these glaciers are available only in the MEaSUREs dataset and the dataset presented here.

The MEaSUREs dataset suggests that The Moscow University Glacier also showed uneven retreat rates between 1996 and 2021. The western flank of the main trunk retreated at 690 ± 40 m/year, the central portion at 270 ± 30 m/year, and the eastern flank at 170 ± 30 m/year. However, when compared to the ESA’s dataset, we conclude that between 1996 and 2017, the eastern flank retreated at 80 ± 30 m/year, the central part remained stable, and the western part retreated at 170 ± 50 m/year, while the major retreat occurred between 2017 and 2021. Specifically, the western flank retreated at 1.4 ± 0.3 km/year, the central portion at 480 ± 60 m/year, and the eastern flank at 510 ± 50 m/year. MEaSUREs, ESA’s, and neural network-based datasets all suggest that the Totten Glacier’s central main trunk exhibited temporally uneven retreat. Between 1999 and 2013, it remained stable, showing no retreat. However, between 2013 and 2020, it advanced inland at a dramatic rate of 2.7 ± 0.2 km/year, which is consistent with the values reported in Ross *et al*.^48^. These observations highlight the spatial variability in retreat rates, not only across different glaciers, but also along the main trunk of individual glaciers, emphasizing the influence of tidal forcing and local dynamics on grounding line behavior.

In the Amundsen Sea Embayment, the Pine Island Glacier exhibited temporally uneven retreat. Between 1999 and 2011, the glacier retreated at a rate of 2.3 ± 0.1 km/year, but starting in 2011, the retreat rate significantly slowed to 150 ± 50 m/year until 2021, which is consistent with Milillo *et al*. (2017), who noticed the same retreat pattern. The grounding live over the region, close to the eastern end of the Thwaites Glacier, commonly referred in literature as Thwaites Butterfly^51,65,66^ displayed spatially uneven retreat between 2011 and 2020. However, when spatially averaged (i.e. when considering the overall grounding line retreat rate across the entire grounding zone of each glacier, rather than focusing only on areas of fastest retreat), the grounding line retreated at a rate of 630 ± 80 m/year. During the period of 2011–2020, the main trunk of Thwaites Glacier experienced a more moderate retreat of 470 ± 50 m/year, while the Haynes Glacier retreated at a slower rate of 210 ± 70 m/year, which is also consistent with previous studies^42,56^. The integration of our DInSAR dataset with a single historic grounding line record from the MEaSUREs dataset enables the calculation of long-term retreat rates, which is essential for assessing the stability of glaciers.

## Data Availability

No custom code has been used.
